# Characteristics of COVID-19 cases with breakthrough infection in the governorate of Sousse, Tunisia

**DOI:** 10.1093/eurpub/ckac131.098

**Published:** 2022-10-25

**Authors:** S Ben Fredj, R Ghammem, N Zammit, A Maatouk, N Hadded, H Laadhari, J Maatoug, H Ghannem

**Affiliations:** 1 Department of Epidemiology, University Hospital Farhat Hached, Sousse, Tunisia; 2 Faculty of Medicine of Sousse, University of Sousse, Sousse, Tunisia; 3 Regional Health Office of Sousse, Ministry of Health, Sousse, Tunisia

## Abstract

**Introduction:**

In early 2021, Tunisia implemented a national COVID-19 strategy of vaccination aimed at disease elimination. In this study, we aimed to investigate the characteristics of the breakthrough COVID-19 infection in the governorate of Sousse, Tunisia.

**Methodology:**

We conducted a cross-sectional study including all post-vaccination COVID-19 cases from March 2021 to August 2021. We collected data via 15-minute telephonic call interviews. We estimated the specific incidence rates (SIR) of confirmed cases by vaccine type and expressed them as cases per 100 000 inhabitants. Statistical analyzes were carried out using anonymous and codified Excel tables and SPSS 20.

**Results:**

Overall, we included 618 cases of breakthrough COVID-19 infection. The majority were female (sex-ratio=0.8), and the average age of the overall cases was 55.7±14.5 years (range:19-91). Nearly half (49%) of participants had comorbidities, 19.6% were healthcare workers, and 17.9% were smokers. The majority of cases (70%) received at least one dose of Pfizer vaccine followed by CORONAVAC (15.6%; n = 96). Nevertheless, we found a higher incidence rate of COVID-19 among those vaccinated with SPUTNIK V (SIR=1551.2) followed by SINOPHARM (SIR=823.7). Fifty-eight percent of patients reported a poor adhesion to preventive measures, whereas 38.6% reported high respect for the preventive measures. COVID-19 infection led to hospitalization in 8.1% of cases, hospitalization in intensive care units in 2.1% of cases, and death in 1.8% of cases.

**Conclusions:**

The findings of our study highlight the low rate of severe cases of COVID-19 among the vaccinated population. Furthermore, we found a discrepancy in the effectiveness of vaccines in the prevention of transmission potential. Yet, many factors could influence the transmission and the severity of COVID-19 breakthrough infection from one region to another.

**Key messages:**

• low rate of severe cases of COVID-19 among the vaccinated population.

• discrepancy in the effectiveness of vaccines in the prevention of transmission potential.

